# RUN(X) out of blood: emerging RUNX1 functions beyond hematopoiesis and links to Down syndrome

**DOI:** 10.1186/s40246-023-00531-2

**Published:** 2023-09-05

**Authors:** Esteban J. Rozen, Christopher D. Ozeroff, Mary Ann Allen

**Affiliations:** 1 Crnic Institute Boulder Branch, BioFrontiers Institute, University of Colorado Boulder, 3415 Colorado Ave., Boulder, CO 80303 USA; 2 Linda Crnic Institute for Down Syndrome, University of Colorado Anschutz Medical Campus, 12700 East 19th Avenue, Aurora, CO 80045 USA; 3 Department of Molecular, Cellular and Developmental Biology, University of Colorado Boulder, 1945 Colorado Ave., Boulder, CO 80309 USA

**Keywords:** RUNX1, Trisomy 21, Down syndrome, Transcription, Alternative splicing

## Abstract

**Background:**

RUNX1 is a transcription factor and a master regulator for the specification of the hematopoietic lineage during embryogenesis and postnatal megakaryopoiesis. Mutations and rearrangements on *RUNX1* are key drivers of hematological malignancies. In humans, this gene is localized to the ‘Down syndrome critical region’ of chromosome 21, triplication of which is necessary and sufficient for most phenotypes that characterize Trisomy 21.

**Main body:**

Individuals with Down syndrome show a higher predisposition to leukemias. Hence, RUNX1 overexpression was initially proposed as a critical player on Down syndrome-associated leukemogenesis. Less is known about the functions of RUNX1 in other tissues and organs, although growing reports show important implications in development or homeostasis of neural tissues, muscle, heart, bone, ovary, or the endothelium, among others. Even less is understood about the consequences on these tissues of RUNX1 gene dosage alterations in the context of Down syndrome. In this review, we summarize the current knowledge on RUNX1 activities outside blood/leukemia, while suggesting for the first time their potential relation to specific Trisomy 21 co-occurring conditions.

**Conclusion:**

Our concise review on the emerging RUNX1 roles in different tissues outside the hematopoietic context provides a number of well-funded hypotheses that will open new research avenues toward a better understanding of RUNX1-mediated transcription in health and disease, contributing to novel potential diagnostic and therapeutic strategies for Down syndrome-associated conditions.

## Introduction

### RUNX genetics and functions

**Fig. 1:**
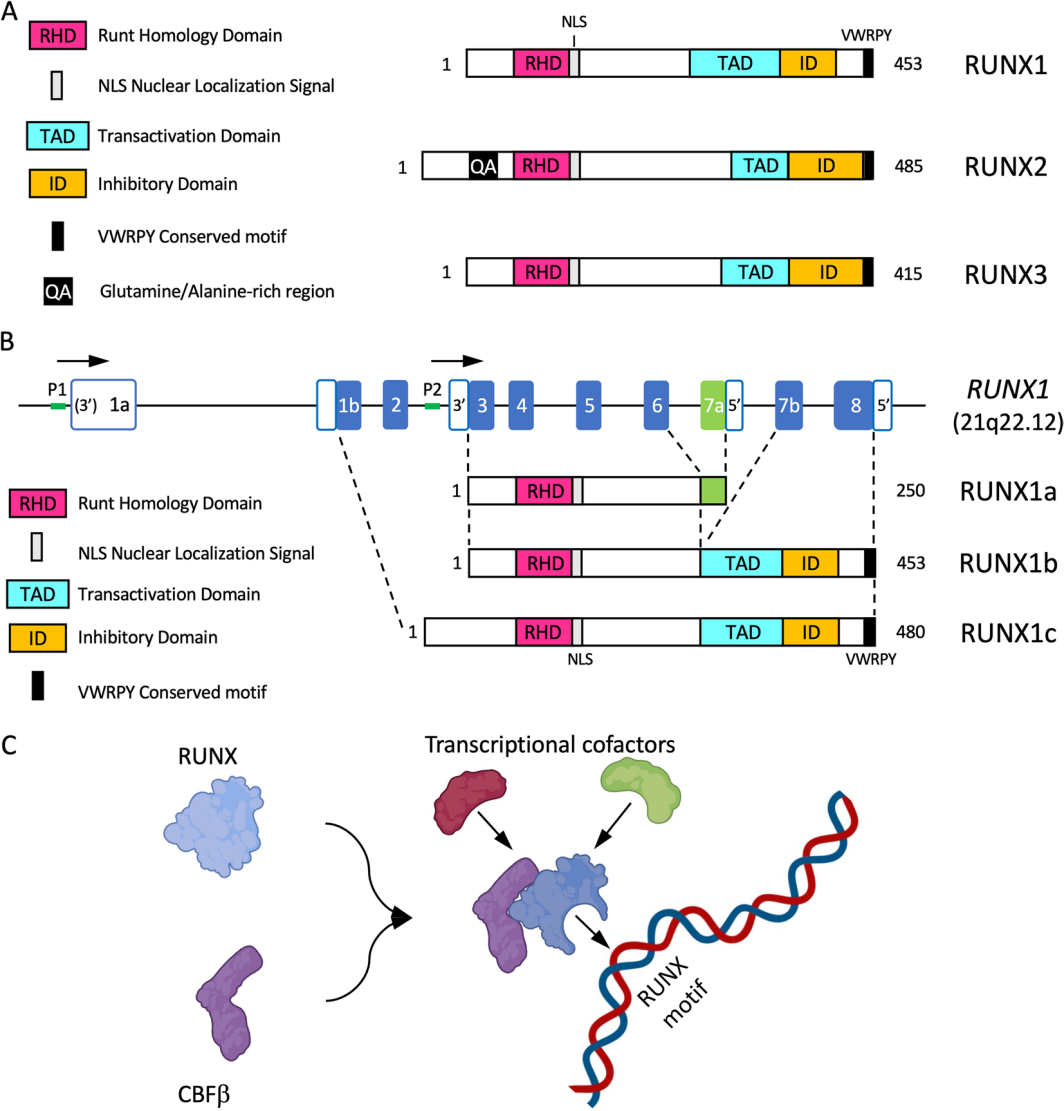
**A.** Domain organization of the RUNX family of transcription factors. General domain architecture of the mammalian RUNX family members. **B.** RUNX1 exon organization and major alternative splicing isoforms. Major RUNX1 variants produced by alternative promoter/splicing mechanisms (see main text and BOX for details). **C.** RUNX-CBFβ form heterodimers to interact with DNA and regulate transcription. Heterodimerization of RUNX TFs with CBFβ is required for enhanced DNA binding, stability and interaction with other transcriptional regulators, leading to induction or repression of target genes

The RUNX family of transcription factors (TFs) play critical functions during development and adult organ homeostasis and are responsible for several human diseases. Runt-related genes (RUNX-1, -2, -3 and CBFβ) are members of the core-binding factor (CBF) family, and participate in a wide array of processes in several cell types and tissues. The three RUNX genes exhibit a highly conserved genomic organization across metazoans (Fig. 1A; reviewed in [1]). In vertebrates, RUNX genes are characterized by the presence of two alternative promoters, P1 (distal) and P2 (proximal) [2, 3] (Fig. 1B). RUNX TFs also feature an evolutionarily conserved Runt homology domain (RHD) in their N-terminal half [3] (Fig. 1A). The RHD is responsible for interaction with the RUNX-cognate DNA motif-PyGPyGGTPy-[4, 5], but also mediates protein–protein interactions [6, 7] and harbors a nuclear localization signal [8, 9]. A transactivation domain (TAD) and an inhibitory domain (ID) are found in the C-terminal portion, together with a terminal VWRPY motif, which binds the Groucho/TLE (Transducin-Like Enhancer of split) family of transcriptional corepressors [10–12]. Heterodimerization of RUNX with CBFβ is required to allosterically enhance the complex stability and affinity for DNA, leading to induction or repression of target genes [13–16] (Fig. 1C).

Recent years have seen significant progress in our understanding of the functions performed by RUNX1 in several developing and postnatal mammalian tissues, a topic that has been recently covered elsewhere [17]. RUNX1, also known as Acute Myeloid Leukemia 1 protein (AML1), is widely considered the master regulator of developmental hematopoiesis because it has a critical function in the specification of the hematopoietic lineage during embryogenesis, while also playing essential roles in postnatal tissue homeostasis and in hematological malignancies.

Noteworthy, in humans, the *RUNX1* gene is localized to band q22.12 of chromosome 21, which is triplicated in individuals with Trisomy 21 (T21). Hence, an extra copy of *RUNX1* has been proposed to play relevant roles in some of the many phenotypic alterations associated with Down syndrome (DS). In addition to alternative promoter usage, in humans, *RUNX1* mRNA splicing gives rise to multiple isoforms, of which RUNX1c (from P1), RUNX1a and RUNX1b (from P2) are the most abundant and best characterized [18, 19] (Fig. 1B). While RUNX1b and RUNX1c are functionally identical, RUNX1a is a shorter variant lacking the transactivation and inhibitory domains (Fig. 1B). For that reason, RUNX1a has been shown to have an antagonic, pro-oncogenic function (see BOX). More importantly, as reported by Komeno et al*.*, the alternative splicing of exon 7a that gives rise to RUNX1a is primate-specific and thus, mice do not naturally express the RUNX1a isoform [19]. In summary, because mouse models of Down syndrome lack the RUNX1a isoform, which may be critical to T21-associated leukemogenesis, they may not be the best system to study RUNX1 function in Down syndrome (Fig. 1B and BOX [19–36]).
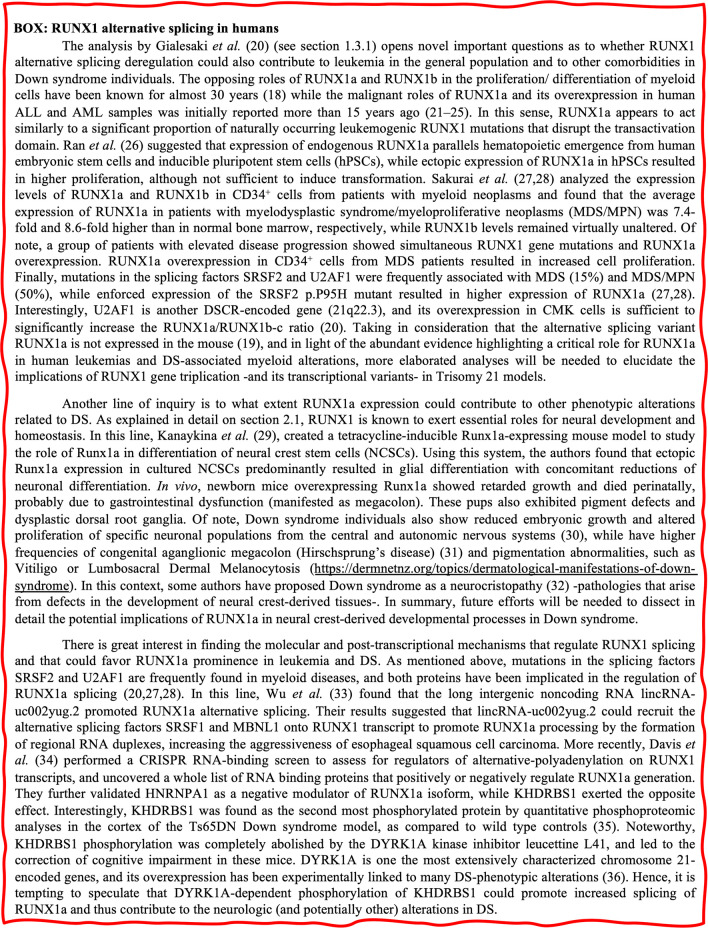


RUNX1 activities in normal megakaryopoiesis and hematopoietic stem cell maintenance, and their relations to hematological diseases, have been extensively studied for many years and, therefore, will not be covered in the present review. On that regard, for additional information refer to the very complete literature published in recent years (e.g., [37, 38]. Nevertheless, DS individuals show a disproportionately higher predisposition to specific hematological malignancies, and RUNX1 overexpression has been proposed to contribute to such dysregulation. Therefore, we will briefly touch on the DS-specific hematological implications of RUNX1. On the other side, much less is known about the functions of this transcription factor in other developmental and homeostatic processes, and how these could contribute to DS comorbidities, which will be the major topics of the following sections.

### RUNX1 in leukemia

**Fig. 2:**
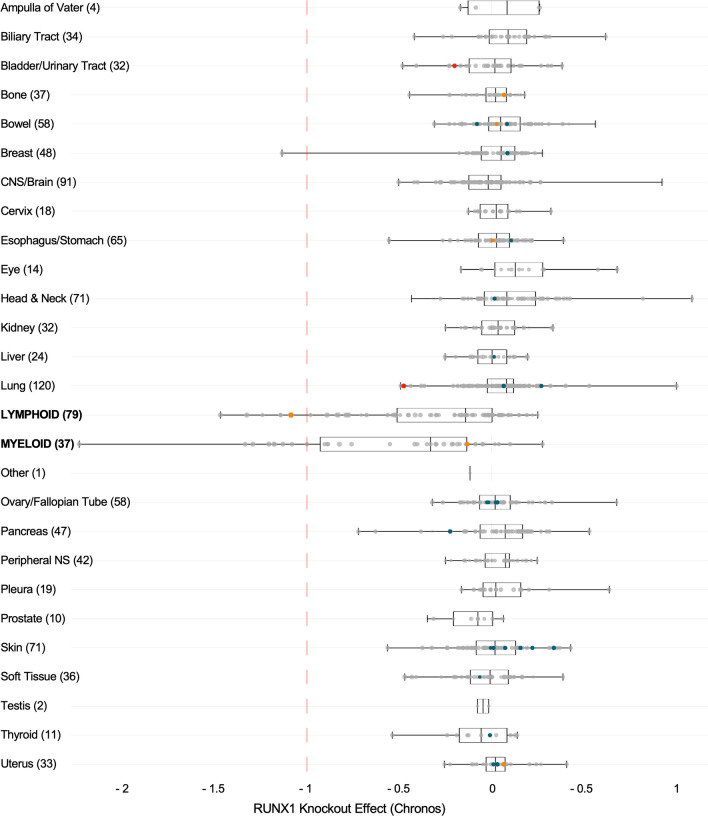
RUNX1 dependency across cancer cell lines from the Cancer Dependency Map portal (depma.org). The Chronos dependency score is based on data from a cell depletion assay. A lower score indicates a higher probability that the gene (RUNX1) is essential for a given cell line. A score of 0 indicates a gene is not essential, − 1 is comparable to the median of all pan-essential genes. Note that a significant proportion of Lymphoid and particularly Myeloid cancer cell lines—but not other cancer types- require RUNX1 expression for survival, as suggested by a Chronos dependency score below − 0.5. Importantly, RUNX1 dependency across Lymphoid and Myeloid cancer cell lines is significantly correlated to the level of RUNX1 mRNA expression in each cell line (not shown)

Mutations of *RUNX1* are the underlying cause of familial platelet disorders that precede several myeloid malignancies. Somatic mutations and chromosomal rearrangements involving *RUNX1* loss-of-function or changes of its transcriptional activity are frequently observed in myelodysplastic syndrome and leukemias of myeloid and lymphoid lineages, such as acute myeloid leukemia (AML), acute lymphoblastic leukemia (ALL), and chronic myelomonocytic leukemia (CMML). This topic is very well understood and therefore, for additional information refer to the excellent available reviews on this [39, 40]. On the other hand, recent studies suggest that wild-type *RUNX1* is also required for growth and survival of certain types of AML cell lines [41]. Similarly, Gialesaki et al*.* observed a strong RUNX1-dependency in DS-associated myeloid leukemia (ML-DS) cell lines [20], which is also seen for many myeloid and lymphoid cancer cell lines from the Cancer Dependency Map portal (https://depmap.org) (Fig. 2).

### Down syndrome and hematopoietic disorders

Newborns with Down syndrome are predisposed to a variety of hematopoietic abnormalities, ranging from relatively benign, such as neutrophilia and macrocytosis, to more severe transient myeloproliferative disorders (TMD). In most cases, these abnormalities resolve in the first few months/years of life. Nevertheless, about 10% of DS-associated TMD patients progress into acute megakaryoblastic leukemia (AMKL). AMKL in individuals with Down syndrome is heavily associated with acquired GATA1 mutations that result in the dominant expression of a short isoform, collectively referred to as GATA1s (short) mutations [42]. GATA1s is a natural alternative splice variant lacking the transactivation domain [42]. Contrary to the full-length variant, GATA1s fails to repress pro-oncogenic MYC expression and the pro-proliferative E2F transcription network, constituting a major mechanism of leukemic transformation [43–46]. Thus, DS children have a significantly higher risk of developing leukemia [47], while showing a dramatically reduced frequency of solid tumors [48]. Children with T21 have ~ 500-fold higher chances of developing AMKL, which is relatively rare in the general population, while the risk of pediatric acute lymphoblastic leukemia (ALL) is 20 to 30-fold greater in DS kids [47]. Several genes lying within the so-called Down syndrome critical region (DSCR) at 21q22—including *RUNX1* and *DYRK1A*—are thought to contribute to such enhanced risk to leukemias [49].

#### RUNX1 in DS-associated leukemia

While RUNX1 is an etiologic factor in AMKL [50], there is conflicting evidence regarding RUNX1 in ML-DS. In the first place, most of the characterized RUNX1 mutations/translocations result in loss of function, suggesting that wild-type RUNX1 plays a tumor-suppressive role. In ML-DS, RUNX1 is not usually mutated. Therefore, overexpression of wild-type RUNX1 in DS cells (due to an extra copy of the gene) is predicted to decrease, rather than augment, the risk of leukemia. Results from several in vivo DS mouse models seem to indicate that RUNX1 is not the cause for the observed abnormal hematopoiesis, myeloproliferative disease or leukemia [51–53]. On one hand, the Ts65Dn murine model of Down syndrome develops persistent macrocytosis and myeloproliferative disease (characterized by thrombocytosis, megakaryocyte hyperplasia with dysplastic morphology, and myelofibrosis). Restoring *RUNX1* gene dosage to disomic levels (Ts65Dn/Runx1^+/±^) did not rescue this phenotype [51]. Carmichael et al*.* [52] observed that the Ts1Cje DS mouse model—also trisomic for RUNX1—has mild defects in mature blood cells, including macrocytosis and anemia, but do not develop TMD or leukemia, even in the context of a Gata1s mutation. Finally, the Tc1 mouse model of Down syndrome also exhibits macrocytic anemia and increased extramedullary hematopoiesis [53, 54]. Moreover, enhanced megakaryopoiesis was seen by concomitant GATA1s mutation in these mice, but not resulting in leukemia or TMD [53]. Noteworthy, the Tc1 model is trisomic for approximately 80% of the genes encoded on Hsa21, but not for RUNX1, ruling out its involvement in the observed Tc1 hematopoietic alterations, but allowing for the speculation of whether its triplication could still lead to a more aggressive myeloproliferative disease in this paradigm. These data are consistent with a role for RUNX1 in the potentiation of megakaryopoiesis [51].

A cooperative functional interaction between wild type GATA1 and RUNX1 is known to drive normal megakaryocytic differentiation [55, 56], and this interaction seems to be conserved in the GATA1s pre-leukemic mutant version in DS-AMKL cells [56]. Using transchromosomic mouse embryonic stem cells (ESCs) bearing an additional copy of human chromosome 21 (HSA21), De Vita, et al*.* [57] found that an extra copy of RUNX1 caused an increase in Tie-2/c-Kit levels, partially contributing to the disturbance of early hematopoiesis in DS. More recently, Vukadin et al*.* [58] proposed a role for another chromosome 21 gene, SON, as a transcriptional repressor of RUNX1 expression and subsequent megakaryocytic differentiation. They showed that SON is aberrantly overexpressed in AMKL, while depletion of SON markedly increased megakaryocytic gene expression and differentiation. In line with this, Bourquin et al*.* [59] previously reported SON as the second most upregulated chromosome 21-encoded transcription factor in DS-AMKL samples (vs. typical AMKL). Interestingly, they also indicated that RUNX1 expression in DS-AMKL patients is decreased, despite its increased gene dosage. Thus, since RUNX1 is essential for normal megakaryopoiesis, reduction of RUNX1 expression by SON in DS-AMKL could contribute to impaired megakaryocytic differentiation. Work from two other groups recently showed that the RUNX1 gene is significantly hypermethylated in neonatal blood samples from DS newborns [60, 61]. Altogether, several pieces of evidence, including data from 3 different murine models of DS, appear to suggest that RUNX1 triplication might not play a crucial role in DS-associated leukemia, but perhaps in milder phenotypes related to megakaryopoiesis and erythropoiesis.

Despite of this, a recent report has provided important evidence challenging the idea that RUNX1 overexpression may not play a role in leukemia. Gialesaki et al*.* [20], uncovered a strong and specific RUNX1-dependency in ML-DS and non-DS AMKL cells. They further demonstrated that expression of the short RUNX1a splicing isoform (Fig. 1B and BOX), which lacks the transactivation domain, is elevated in patients with ML-DS. Mechanistic studies using murine ML-DS models and patient-derived xenografts revealed that during leukemogenesis, elevation of RUNX1a displaced the full-length RUNX1c variant from its endogenous binding sites, inducing oncogenic programs through direct interaction with MYC/MAX, and thus, synergizing with the pathognomonic Gata1s mutation. Hence, overexpression of the shorter alternative splicing variant RUNX1a, with opposed functions to the full-length protein, could indeed contribute to the leukemic phenotype in specific DS cell types. Since RUNX1a is not expressed in rodents [19], interpretation of experiments on RUNX1 functions from mouse models of Down syndrome and their extrapolation to humans should be done with great caution.

#### RUNX1 in other DS-related phenotypes

As previously discussed in section "RUNX1 in DS-associated leukemia.", the implications of RUNX1 in Trisomy 21 have been mostly studied in the context of hematological malignancies. Nevertheless, several groups have started to shed light into potential functions of this pleiotropic transcription factor in other DS-related alterations. Mollo et al*.* [62] re-analyzed mouse gene expression data obtained after over-expression of individual Hsa21 genes (GEO GSE19836 [63]) and saw that the transcription factor Runx1 can induce upregulation of extracellular matrix (ECM) genes. Moreover, the ECM was consistently among the most affected GO categories by gene set enrichment analysis (GSEA) from public data (including experiments with either RUNX1 upregulation or downregulation). Interestingly, they quantified that ~ 80% of ECM genes overexpressed in T21 hearts had RUNX1 consensus sequences. Furthermore, they saw that in T21 human fetal fibroblasts there is increased expression of both RUNX1 and several ECM genes, which decreased upon RUNX1 silencing. These results suggest potential implications for RUNX1 in ECM alterations as an underlying cause for DS-related congenital heart defects [64], Hirschsprung’s disease [65] and/or pulmonary hypertension [66], among other ECM-mediated pathologic conditions.

Halevy et al*.* [67] characterized five independent DS-derived embryonic stem cell lines to show that differentiation of neural progenitor cells (NPCs) displayed increased apoptosis, with upregulation of mitochondrial apoptotic-related genes and downregulation of forebrain developmental genes. Analysis of differentially expressed genes (DEGs) suggested that RUNX1 overexpression may disrupt different molecular pathways during neural development in DS-NPCs. RUNX1 disruption by genome editing resulted in reduced apoptosis and neuron migration. In line with these findings, Liu et al*.* [68] reported that RUNX1 expression levels in DS-iPSCs were significantly higher than in controls and correlated with impaired mitochondrial functions and increased apoptosis, while inhibition of RUNX1 expression improved the mitochondrial function in DS-iPSCs. Altogether, the roles of this important transcription factor in DS-associated alterations are just starting to emerge, warranting future efforts to clearly elucidate the implications of RUNX1 and its alternative splicing regulation on the different pathophysiological features of Trisomy 21.

## RUNX1 beyond blood

### Neurodevelopment

Although very little is known about the roles of RUNX1 triplication in DS-related neurological alterations [67], several groups have studied the functions of this transcription factor in normal neural development and homeostasis. Theriault et al*.* [69] showed Runx1 expression in specific neurons of the embryonic central and peripheral nervous systems of the mouse, including cholinergic branchial and visceral motor neurons of the hindbrain, certain spinal cord somatic motor neurons, and in nociceptive and mechanoreceptor neurons from the trigeminal and vestibulocochlear ganglia. Runx1 knock-out resulted in defects on specific sensory neurons in the trigeminal and vestibulocochlear ganglia, as well as loss of cholinergic neurons in the hindbrain mantle layer. In a follow-up study [70], these authors also showed Runx1 expression in proliferating cells of the olfactory epithelium, and that in vivo Runx1 impairment resulted in premature and ectopic olfactory receptor neuronal differentiation. Ectopic Runx1 expression increased cell proliferation of olfactory and cortical neural progenitors. These observations are further supported by the fact that RUNX1 is induced in a subpopulation of adult neural stem or progenitor cells NS/PCs after brain injury in mice [71]. Furthermore, Runx1 downregulation in neurosphere cultures of adult mouse subventricular zone NS/PC inhibited proliferation without affecting differentiation. Noteworthy, Runx1 overexpression induced adult NS/PC differentiation predominantly toward a neuronal lineage, without reducing proliferation. On the other hand, Fukui et al*.* [72] showed that overexpression of Runx1 in cultured adult hippocampal precursor cells reduced proliferation, increased survival (reduced apoptosis), and enhanced neuronal differentiation, while slightly reducing dendritic morphology and complexity. Finally, Shrestha et al*.* [73] demonstrated that Runx1 controls the diversity of auditory sensory neurons—spiral ganglion neurons (SGNs)—in mice. The authors found that Runx1 is enriched in Ib/Ic SGN precursors by late embryogenesis, while embryonic loss of Runx1 resulted in greater numbers of Ia identity at the expense of Ib/Ic fates. Similarly, during mouse embryonic development, Runx1 is expressed in most nociceptor neurons, but becomes restricted to Ret^+^ nociceptors in adult mice [74]. In this line, Huang et al*.* [75] also reported that the Runx1/CBFβ transcriptional complex is essential for NGF/TrkA-dependent differentiation of nonpeptidergic nociceptors of the dorsal root ganglion (DRG). Of note, Kramer et al*.* [76] had previously characterized a role for Runx1 in diversification of the DRG sensory neurons. As a summary, while there are cell context-specific differences and interpretations, all these studies confirm that RUNX1 promotes survival, proliferation and/or differentiation of several neuronal populations. Importantly, except for the work from Halevy et al*.* [67], all of these reports were performed in mouse models that do not express the Runx1a isoform. For that reason, it is worth remembering that ectopic expression of Runx1a in cultured mouse Neural Crest Stem Cells undergoing neural differentiation predominantly resulted in glial differentiation with concomitant reductions of neuronal fates [29], while Runx1a-overepressing pups exhibited dystrophic DRGs and megacolon, presumably due to impaired survival or migration of the enteric nervous system precursors along the gastrointestinal tract (Hirschsprung’s disease). These results would suggest that contrary to murine Runx1, Runx1a inhibits neuronal survival, proliferation and/or differentiation, which is in agreement with the idea that RUNX1a acts as an endogenous antagonist of RUNX1b/c transcriptional functions [20, 21]. Therefore, the potential species-specific differences in RUNX1 alternative splicing and function during the development and differentiation of neuronal populations remains to be elucidated, a topic of profound relevance, as many of the above-mentioned RUNX1-dependent neurodevelopmental processes are frequently altered in Down syndrome individuals and experimental models (reviewed in [77]), such as the olfactory neuroepithelium [78, 79], subventricular zone neurogenesis [80, 81], the NGF-dependent cholinergic system [82], motor-neurons [83] or the cochlear sensory neurons [84, 85], among many others.

### Bone and cartilage

RUNX1 functions in bone and cartilage development and homeostasis are very well established and have recently been reviewed elsewhere [86]. Therefore, we will only briefly summarize the most relevant literature and focus on potential links to Down syndrome-associated conditions. Initial reports in the 2000’s indicated that Runx1 may contribute to the early stages of murine craniofacial bone development and skeletogenesis, while continuing to function in the progenitor cells of tissues that support bone formation in the adult [87, 88]. Since then, a wealth of literature has confirmed and explored in more detail the mechanisms by which RUNX1 modulates both chondrogenesis and osteogenesis. In this line, Soung et al*.* [89] examined the potential role of Runx1 in osteoclast formation and function. To this end, the authors deleted Runx1 expression in myeloid osteoclast precursors (Runx1^f/f^; CD11b-Cre) and observed significant loss of femoral trabecular and cortical bone mass compared with control littermates. These and other experiments indicated that Runx1 expression in preosteoclasts negatively regulates osteoclast formation and activity, and contributes to overall bone mass. In parallel, mice with chondrocyte-specific deletion of Runx1 (Runx1^f/f^; Col2α1-Cre) manifested alterations in cartilage formation, reduced bone density, and an osteoporotic phenotype, suggesting that Runx1 is required for both chondrogenetic and osteogenetic gene expression programs [90]. Moreover, Runx1 ablation in osteoblast precursors and differentiating chondrocytes (Runx1^f/f^; Osx-Cre mice) resulted in an osteoporotic phenotype and decreased bone density in the long bones and skull compared to controls [91]. The authors also found that Runx1 upregulates the expression of multiple bone-specific genes and plays an indispensable role in bone formation and homeostasis for both trabecular and cortical bones. Furthermore, Tang et al*.* [92] generated a doubly transgenic mesenchymal progenitor-specific (Runx1^f/f^; Twist2-Cre) and osteoblast-specific (Runx1^f/f^; Col1α1-Cre) double conditional knockout (Runx1 dCKO) mouse model. Runx1 dCKO resulted in decreased osteogenesis and increased adipogenesis, while molecular analysis demonstrated that Runx1 maintains adult bone homeostasis though up-regulating Bmp7/ATF4 and WNT/β-Catenin signaling pathways. Similarly, osteoclast-specific conditional knock-out of Runx1 (Runx1^f/f^; LysM‐Cre mice) compromised murine fracture healing due to progressive woven bone loss and hindered remodeling of the cartilage [93]. Finally, in an experimental model of osteoarthritis using Runx1^f/f^; Col2α1-Cre mice, Zhou et al*.* [94] observed that chondrocyte-specific Runx1 knockout aggravated cartilage destruction, decreased chondrocyte proliferative capacity and enhanced loss of bone matrix.

People with Down syndrome are predisposed to skeletal deficits, including lower body height, reduced mineral density in the bone, and increased frequency of early-onset osteoporosis, with significant alterations in skeletal and craniofacial development, bone morphology and homeostasis, and age-related bone loss and fragility (reviewed in [95]). However, the genetic origins of DS-skeletal phenotypes remain unclear, although several pieces of evidence suggest a contributing role for the chromosome 21-encoded gene *DYRK1A* [95–97]. Moreover, alterations in chondrogenesis [98] and a predisposition to Down syndrome-associated arthritis [99] have been reported. Surprisingly, the potential implications of *RUNX1* triplication or changes in its alternative splicing on bone/cartilage homeostasis in the context of Down syndrome have not been reported. Noteworthy, and as previously pointed, newborn mice overexpressing Runx1a were smaller than the control littermates [29], again suggesting a potential negative regulatory function over the longer isoforms Runx1b/c during embryonic development of the murine skeleton. This observation, together with the very well-known roles played by RUNX1 in bone and cartilage biology, and the important skeletal alterations seen in Down syndrome individuals warrant for future efforts to elucidate the interconnections linking *RUNX1* gene triplication to bone homeostasis and disease in Trisomy 21.

### Heart

During embryonic development, RUNX1 is expressed in the mesenchymal tissue of the heart and vascular tissues [100]. Runx1 KO mice have an underdeveloped coronary plexus and smaller ventricular free wall vessels, accompanied by changes in heart structure, such as ventricular septal defects and thinner myocardium [101]. Neonatal cardiomyocytes exhibited significantly increased *Runx1* mRNA and other markers of cardiac differentiation (Myh7, Nkx2.5, Dab2, Destrin) than adult counterparts [102]. Górnikiewicz et al*.* [103] performed global DNA methylation profiles in whole murine hearts and found that promoter regions with increased DNA methylation at post-natal day 7 (compared to day 1) were significantly enriched for Runx1 binding motifs and included genes critical for heart maturation and muscle development. In the adult heart, RUNX1 is silenced, but becomes upregulated under cardiac pathologic conditions (reviewed by [104]), such as ischemic cardiomyopathy [105], chronic dilated cardiomyopathy [106, 107] and in animal models of diabetic cardiomyopathy and pressure overload [108, 109]. Recent work from Loughrey’s group shows that Runx1 ablation [107] or pharmacologic inhibition [110] has a protective effect on heart function after myocardial infarction. More recently, Swift et al*.* [111] reported that Runx1 plays a critical role in cardiomyocyte ploidy dynamics and cell division, in both developmental and injury contexts. Overall, RUNX1 is required for the proper development of cardiovascular tissues and its expression correlates with disease in the adult heart; therefore, it is somewhat unexpected that RUNX1 gene triplication has not been widely associated to Down syndrome-related congenital heart disease. In this context, changes in composition of the extracellular matrix (ECM) are suggested to contribute to heart defects in Trisomy 21 [112]. Moreover, Conti et al*.* [113] analyzed the transcriptional profile of human fetal hearts from DS fetuses and found that genes coding for ECM proteins are the most over-represented among the upregulated ones. Finally, Mollo et al*.* [62] found that about 80% of the ECM genes upregulated in DS fetal hearts had RUNX1 consensus sites in their promoter regions, while experimentally confirmed the role for RUNX1 as a main controller of ECM gene expression in T21 fetal fibroblasts.

About 50% of newborns with DS have some kind of congenital heart disease (CHD), including ~ 43% atrioventricular septal defects (AVSD), ~ 32% ventricular septal defects (VSD), ~ 19% isolated secundum atrial septal defects (ASD), ~ 7% isolated persistent patent ductus arteriosus (PDA) and ~ 6% tetralogy of Fallot (TOF), among others [114, 115]. Historically, this has been one of the major causes of reduced life expectancy in individuals with Trisomy 21, whereas advances in diagnosis, treatment, and surgery for CDH in DS individuals has led to a dramatic improvement in survival over the last 60 years [116]. Yet, the genetic and molecular mechanisms underlying DS-CDH are only starting to emerge (reviewed in [117, 118]). In this sense, several chromosome 21-encoded genes have been associated with cardiovascular defects, such as *DYRK1A*, *COL6A1-2*, *KCNJ6* and *RCAN1* (reviewed in [118]). Altogether, more research is needed to clearly define the implications of RUNX1 overexpression in DS heart development and homeostasis, which could allow more precise interventions to correct the genetic programs underlying DS-CHD.

### Muscle

Initial reports suggested that Runx1 is not detected during embryonic muscle development [100, 119] or in the healthy adult muscle [120]. Nevertheless, Runx1 becomes highly upregulated in muscles exposed to myopathic damage. Hence, similarly to what is observed in the heart (see previous section), RUNX1 expression is significantly increased in samples of muscle dystrophies, including mouse models of Duchenne muscular dystrophy (DMD) [121] and amyotrophic lateral sclerosis [122], myopathy patients—including DMD, Emery-Dreifuss Muscular Dystrophy and Acute Quadriplegic Myopathy—[123], as well as in cardiotoxin-treated muscles [124] and during ischemia reperfusion-induced muscle injury [125]. Nevertheless, studies on the role of Runx1 in myoblasts have reached conflicting conclusions [126–128]. To address this, Umansky et al*.* [129, 130] generated mice specifically lacking Runx1 in the muscle (Runx1^f/f^; Myf5-cre) and found that Runx1 expression is switched on in response to muscle damage and cooperates with the MyoD and AP-1/c-Jun transcription factors during muscle regeneration by preventing premature myoblasts differentiation. Thus, Runx1-deficient primary myoblasts differentiated prematurely, reducing the number and size of regenerating myofibers and impairing muscle regeneration. Overexpression of Runx1 in C2C12 cells inhibited myogenic differentiation, while promoting myoblast proliferation [125]. Consistent with this, they found that Runx1 expression correlated with that of Pax7 in undifferentiated satellite cells, suggesting that Runx1 regulates muscle regeneration by promoting proliferation of satellite cells. These results are consistent with other reports in the context of cardiomyocyte injury and regeneration (i.e., see [111] and section "Heart"). More recently, Stefanowicz et al*.* [131] confirmed that Runx1 is required for myoblast proliferation and subsequent differentiation. Finally, in muscular fibroadipogenic progenitors, Runx1 expression is restricted by miR-206. Runx1 upregulation in these cells induces a transcriptional program that leads to their differentiation into adipocytes, resulting in intramuscular fatty deposits, which are characteristic of muscular dystrophies and aging [132].

Among many other features, Down syndrome individuals are characterized by muscle hypotonia and associated motor and postural deficits [133, 134]. Morphometric analysis demonstrated a larger size of myofibers in the quadriceps muscles of Ts65Dn mouse model of Down syndrome. However, myofibrils were thinner and contained higher amounts of mitochondria and intramuscular fatty deposits (lipid droplets), typical alterations of early aging [135]. Interestingly, Pawlikowski et al*.* [136] reported a failure in satellite (muscle stem) cell expansion in Ts65Dn mice that resulted in impaired muscle regeneration. Ts65Dn satellite cells appeared to accumulate DNA damage, prompting the idea that stem cell dysfunction is a common contributor to multiple Down syndrome phenotypes. Since RUNX1 expression is induced in and required for proliferation of myoblasts in the injured muscle, it is tempting to speculate that in the context of trisomy 21, overexpression of the antagonic RUNX1a isoform could lead to a reduced proliferation and premature differentiation of muscle progenitors. Alternatively, elevated basal/constitutive expression of full-length RUNX1 in satellite cells could potentially result in exhaustion of this population, leading to the observed phenotype. In conclusion, triplication of RUNX1 could be associated with pathological and regenerative states of the muscle, a tissue largely affected in the Down syndrome population. Therefore, future work should address the specific roles of RUNX1 in DS muscle health and disease, with a particular focus on the potential implications of the alternatively spliced variants, not present in previously used murine models.

### Eye/endothelium

Stewart et al*.* [137] reported Runx1 expression in post-mitotic cells of the mouse inner retina at embryonic day 13.5, overlapping with the early amacrine and ganglion cell marker Islet1. More recently, single-cell RNA-seq analyses have confirmed RUNX1 expression in specific retinal ganglion cell subpopulations [138]. Lam et al*.* [139] identified RUNX1 as a gene upregulated in vascular endothelial cells from human Proliferative Diabetic Retinopathy (PDR) fibrovascular membranes (FVMs). In vitro studies using human retinal microvascular endothelial cells (hRMVECs) showed increased RUNX1 RNA and protein in response to high glucose, while RUNX1 inhibition reduced cell migration, proliferation, and tube formation. Immuno-staining assays showed that RUNX1 upregulation is a hallmark of aberrant retinal angiogenesis, later confirmed by others [140, 141]. Finally, the RUNX1 small-molecule inhibitor Ro5-3335 induced a significant reduction of neovascular tufts in oxygen-induced retinopathy models. In a follow-up study, RUNX1 upregulation was proposed to drive epithelial-to-mesenchymal transition (EMT) in primary cultures from human Proliferative Vitreoretinopathy (PVR) membranes. Importantly, topical administration of a Ro5‐3335 nano-emulsion curbed the progression of disease in a novel rabbit model of mild PVR [142]. Furthermore, Whitmore et al*.* [143] suggested that RUNX1 upregulation in PDR fibrovascular membranes is mediated through a TNFα/JNK signaling module. These authors further characterized RUNX1 expression in critical cell types involved in a laser-induced murine model of Choroidal Neovascularization (CNV) and provided data supporting RUNX1 inhibition as a new potential therapy for neovascular age-related macular degeneration [144]. In parallel, Xing et al*.* [145] confirmed RUNX1’s role as a mediator of high-glucose-induced proliferation and migration of hRMVECs. On the other hand, RUNX1 and SMAD3 are required for maintenance of corneal epithelial identity and homeostasis, as their depletion inhibits PAX6 and induces limbal stem/progenitor cells to differentiate into epidermal-like epithelial cells [146]. Finally, Voronov et al*.* [147] reported a role for Runx1/3 during lacrimal gland (LG) morphogenesis, where Runx1 was restricted to the epithelium, with highest levels of expression in ductal and centroacinar cells. Downregulation of Runx1-3 expression abolished LG growth and branching. Noteworthy, RUNX1 is known to play important roles in angiogenesis outside the retina. In this context, Runx1-deficient embryos show imperfect angiogenesis in head, pericardium, and liver [148]. Runx1 loss of function in zebrafish embryos leads to hematopoietic and vasculogenic defects [149]. Runx1 is suggested to directly bind to the *VEGFA* promoter and repress its transcription [150]. At last, Runx1 can induce endothelial cell differentiation and maturation, as well as vascular network formation by repression of *IGFBP3* expression (151). To the best of our knowledge, RUNX1 expression and activity on retinal and lacrimal gland development and function have never been studied in the context of Trisomy 21, a topic that should be taken in consideration toward designing better diagnostic and therapeutic innovations for ophthalmologic alterations of Down syndrome individuals.

People with trisomy 21 manifest a wide range of ophthalmologic features, including strabismus, amblyopia, accommodation defects, refractive error, eyelid abnormalities, nasolacrimal duct obstruction, nystagmus, keratoconus, cataracts, retinal abnormalities, optic nerve defects, and glaucoma. In this regard, for additional information refer to recent reviews that cover this topic in depth [152, 153], while we will only focus on those features that more closely relate to RUNX1 functions in the eye, such us anomalies of the retinal vasculature, optic nerves, or the lacrimal gland (see below). Children with Down syndrome show an increased incidence in retinal abnormalities, with reports ranging from 1.7 to 40% [154, 155]. Williams et al*.* [156] first described a significantly increased density of retinal vessels crossing the margin of the optic nerve head in T21 cases. Sherk and Williams [157] noted an increased number of large vessels crossing the optic disk in DS patients, while Parsa and Almer [158] suggested that the abnormal optic disk vessels in DS eyes may be due to an extra copy of the chromosome 21-encoded gene endostatin, a potent inhibitor of angiogenesis, endothelial cell proliferation, and migration. Optic nerve abnormalities are also frequently associated with T21, with one study reporting a 14% incidence in a cohort of 806 children with Down syndrome [159]. Several other clinical studies have confirmed and extended these observations, characterizing a spectrum of retinal abnormalities in Down syndrome individuals [160–163]. Finally, nasolacrimal duct obstruction (NLDO) is another common congenital finding in DS children, and is caused by different anatomic etiologies, such as membranous obstruction at the valve of Hasner or general stenosis of the duct [164–166].

### Ovary

Ovulation is originated with a peak of luteinizing hormone (LH) from the pituitary, resulting in a transcriptional reprogramming of ovarian granulosa cells leading to structural remodeling of the ovarian follicle and oocyte meiotic maturation. Up-regulation of RUNX1 mRNA and protein was initially detected in preovulatory follicles after human chorionic gonadotropin (hCG) injection in immature rats as well as after the luteinizing hormone surge in cycling animals. Moreover, in primary cultures of rat preovulatory granulosa cells, hCG also induced *Runx1* mRNA expression, while knockdown of Runx1 decreased progesterone secretion and reduced expression of preovulatory signature genes [167], such as *Ptgs2* [168] and *Hapln1* [169]. Altogether, these pieces of evidence suggest that RUNX1 could mediate the LH-dependent shift from estrogen to progesterone production, an essential step in ovulation. More recently, Runx1 was also characterized as a critical female determinant gene, contributing to early sex determination by directing the developmental program for ovarian commitment [170]. In this regard, Runx1 expression is found in early mouse ovaries, but not testes. As stated above, the role of RUNX1 in the ovary goes beyond embryonic development, i.e., in granulosa cells of pre-ovulatory rodent follicles [167, 171]. Similar results were observed in human ovaries [172]. RUNX1 expression is also detected in cumulus cells, a non-luteinizing type of granulosa cells that surround the oocyte, forming the cumulus oocyte complex (COC), released at ovulation [173]. Nicol et al*.* [174] reported that RUNX1 expression fetal ovaries of rainbow trout, turtle, mouse, goat, and human. In mice, pre-granulosa cells express RUNX1 as the gonads differentiate. The authors proposed a RUNX1-FOXL2 cooperative interaction for the identity maintenance of fetal granulosa cells, disruption of which led to masculinization of fetal ovaries. Interestingly, Bridges et al*.* [175] demonstrated that conditional loss of Runx1 in ovarian somatic cells led to increased prevalence of ovarian tumors in aged mice. At younger ages, abnormal follicle-like lesions where abundant, with quiescent granulosa cells and differentiation defects, supporting the idea that Runx1 is essential for granulosa cell differentiation and inhibition of ovarian tumorigenesis. More recently, Dinh et al*.* [176] reported an ovary-specific Progesterone Receptor (PGR)-RUNX1 interaction in 70% of PGR-bound regions, key for the induction of essential ovulatory genes. Finally, the RUNX-binding partner CBFβ was also shown to play a prominent role in ovarian granulosa cells and female fertility [177, 178]. Noteworthy, conditional knock-out of Runx1 in decidual stromal cells—a transient uterine tissue that supports the fetus during pregnancy—leads to defects in uterine angiogenesis, trophoblast differentiation, and vascular remodeling, resulting in fetal lethality during placentation [179].

Initial studies on follicular development in ovaries from Down syndrome girls suggested absence or retardation of follicle growth compared to aged-matched controls. Reductions in the number and size of follicles occurs earlier in life, potentially due to a hormonal imbalance [180]. According to Hsiang et al*.* [181], primary gonadal deficiency is common in DS, progressing from birth to adolescence, and clearly manifest in adult patients (both male and female). Cento et al*.* [182] reported a significantly higher incidence of anovulation and luteal defects in a group of regularly menstruating DS women, while showing reduced estradiol plasma concentrations along the cycle and lower progesterone levels in the mid-luteal phase. In a follow-up study, the authors administered follicle-stimulating hormone (FSH) to a group of normo-ovulatory DS vs. control individuals and found a significant impairment in the ovarian sensitivity to FSH, as measured by estradiol production [183], and potentially contributing to gonadal disfunction in T21 women [184]. Alternatively, Angelopoulou et al*.* [185] suggested that primary gonadal dysfunction in Down syndrome might possibly result from an adrenal gland disorder. Finally, as reviewed by Parizot et al*.* [186], puberty appears to be normal in women with DS and starts at the same average age as in the general population, while women with DS are fertile; however, data have highlighted early menopause in women with DS [187] and a significant reduction of anti-Müllerian hormone [188].

The gene networks underlying developmental and functional alterations in Trisomy 21 ovaries remain virtually unexplored. In summary, the potential functions of RUNX1 during ovarian development and reproduction in the context of Down syndrome remain to be fully elucidated, a field that demands future attention due to the important implications on female endocrine development and fertility of girls and women with Trisomy 21.

### Other tissues

Recent work from several groups has started to address the contribution of RUNX1 in development and/or homeostasis of several other organs, tissues, and cellular functions, which are also altered in Down syndrome individuals and models, again suggesting potential implications for RUNX1 triplication as an underlying cause of such deregulated functions.

#### Nonalcoholic fatty liver disease (NAFLD)

Kaur et al*.* [189] explored the role of RUNX1 in the pathogenesis of non-alcoholic steatohepatitis (NASH). RUNX1 expression was significantly correlated with inflammation, fibrosis, and NASH activity score in patients, while in vitro analyses suggested that steatosis-induced RUNX1 levels lead to upregulation of adhesion and angiogenic molecules in endothelial cells and may be involved in enhancing inflammation and disease severity in NASH. Alternatively, Marcher et al*.* [190] showed that the transcriptional regulators ETS1 and RUNX1 act as drivers of NASH-associated hepatic stellate cell activation and transcriptional plasticity, key aspects of NASH pathophysiology. More recently, Bertran et al*.* [191] analyzed RUNX1 expression in a well-characterized cohort of women with morbid obesity and NAFLD and reported that hepatic RUNX1 expression increases in the first steps of NAFLD but decreases in advanced stages of the disease. Subsequently, these authors applied a systems biology mathematical model that simulates NAFLD pathophysiology concluding that RUNX1 has a high relationship with hepatic injury-liver fibrosis, and a medium relationship with lipotoxicity and insulin resistance [192]. Earlier in vitro studies by Bertrand-Philippe et al*.* [193] using hepatic stellate cells, featured RUNX1 as a critical regulator of tissue inhibitor of metalloproteinase 1 (TIMP1), a key mediator of liver fibrosis [194, 195]. Importantly, the authors observed that overexpression of full-length RUNX1b isoform repressed TIMP1 promoter activity, whereas the truncated RUNX1a isoform and RUNX2 exerted the opposite effect, raising the possibility that a deregulation in RUNX1 splicing, rather than its overexpression, could contribute to a higher NAFLD prevalence in children with Trisomy 21. Finally, Guo et al*.* [196] suggest that RUNX1 induces liver fibrosis progression by directly regulating TGF-β/SMAD signaling.

As reviewed by De Matteo and Vajro [197], the liver is frequently affected in persons with Down syndrome, as evidenced at birth by transient myeloproliferative disorder (TMD)/abnormal myelopoiesis (TAM) [198] and neonatal cholestasis [199], while at later stages by celiac disease-/autoimmunity-related liver diseases [200], and gallstone formation due to gallbladder hypomotility [201]. Noteworthy, hepatic inflammation was also recently described in the Down syndrome mouse model Dp16, which was exacerbated by chronic inflammation with poly(I:C) and reduced by JAK1 inhibition [202]. Valentini et al*.* [203] reported a higher incidence of non-alcoholic fatty liver disease (NAFLD) in 84 children with Trisomy 21 independently of overweight/obese phenotype, in agreement with older reports suggesting an elevated frequency of hepatic steatosis in DS children [204, 205]. In a follow-up study, these authors found a prevalence of 64.3% of liver steatosis in T21 children, which was correlated to the PNPLA3 rs738409 variant and IL6 levels [206]. Altogether, these pieces of evidence point to a plausible role for RUNX1 triplication in the predisposition of individuals with Down syndrome to develop liver diseases, a topic that requires future elucidation.

#### Hair follicle stem cells

Initial studies by Raveh et al*.* [207] found prominent Runx1 expression in epithelial cells of the hair follicle, including the keratin forming layers of the hair shaft and the bulge, where Runx1 is co-expressed with the hair follicle stem cell (HFSC) marker Keratin15. During early hair morphogenesis, Runx1 is also expressed in a discrete dermal mesenchymal layer, while at later stages it is present in a hair cycle-dependent pattern in the dermal papilla. Mice with epidermal conditional knockout of Runx1 displayed structural alterations of the zigzag hair type. In parallel, Soma et al*.* [208] observed intense Runx1 immunoreactivity in the keratinizing area of human anagen hair follicles, and suggested a potential role in transcriptional regulation of the keratin-associated protein hKAP5.1. Subsequent work from Osorio et al*.* [209] showed that keratinocyte-specific conditional Runx1 knock-out impairs HFSC activation, but not their maintenance, proliferation, or differentiation. Adult mutant mice exhibited impaired de novo production of hair shafts and all temporary hair cell lineages, owing to a prolonged quiescent phase of the first hair cycle. In a follow-up report they found that in adulthood, Runx1 plays a direct, non-essential function in promoting anagen onset and HFSC proliferation by blocking the expression of the cyclin-dependent kinase (CDK) inhibitor p21, providing evidence for a role in mouse skin tumorigenesis [210]. Further work from this group showed that Runx1 is required for correct adult HFSC differentiation and skin integrity [211]. Lee et al*.* [212] analyzed in more detail the links between RUNX1 and p21, to find that Runx1 represses transcription of p21 (and other CDK inhibitor genes) in HFSC in vivo, while p21 appears important for the timely onset of quiescence in HFSC. Subsequent studies reported that forced Runx1 expression induces hair degeneration (catagen) and simultaneously promotes a reversible differentiation of bulge SCs toward ‘early progenitor hair germ’ (EP-HG) cell fate, further proposing that changes in endogenous Runx1 levels ensure a proper balance between SC and EP populations during niche restructuring in normal tissue homeostasis [213]. Finally, Li et al*.* [214] showed that Runx1 ablation in the skin epithelium not only delayed stem cell activation and hair cycle progression, as previously seen, but also increased the density of vasculature in the horizontal plexus under the hair germ. The authors propose a model whereby increased vasculature near the HFSC activation zone is inhibitory to stem cell activation and prolongs quiescence by delaying progression from telogen into anagen.

Despite dramatic increases in life span, people with Down syndrome manifest accelerated aging signs, including hair graying, hair loss, and skin wrinkling. Hair loss in DS is typically associated to Alopecia areata [215, 216], an autoimmune condition in which hair follicles are attacked by the immune cells, likely secondary to complex immune dysregulation caused by trisomy of chromosome 21 genes, such as Autoimmune Regulator—AIRE—[217] and 4 interferon receptor genes [218, 219]. Given the well-established implications of RUNX1 in hair follicle stem cell biology, we hypothesize a potential contribution for this transcription factor in the altered hair follicle homeostasis of people with Trisomy 21. In this context, Baars et al*.* [220] showed that RUNX1 acts as a transcriptional regulator of RASGRP1, expression of which is inversely correlated to autoimmunity. Moreover, in patients with autoimmunity, CD4^+^ T cells exhibited reduced RUNX1 and RASGRP1 expression, which was correlated to active inflammation. Similarly, RUNX1 is induced by Influenza A virus infection, acting as a negative modulator of the IFN signaling pathway [221]. Lastly, Zezulin et al*.* [222] observed that RUNX1 plays a role in limiting toll-like receptor 4 (TLR4)-dependent inflammatory response in neutrophils, including type I IFN signaling. In summary, several pieces of evidence indicate a role for RUNX1 as a negative regulator of inflammation and autoimmunity. Thus, we hypothesize that overexpression of the functionally antagonistic RUNX1a isoform in DS immune cells could promote an increased autoimmune response that contributes to Alopecia areata, and other autoimmune reactions associated with Trisomy 21.

#### Pulmonary hypertension (PH)

Immunohistochemistry staining by Levanon et al*.* [100] showed Runx1 expression in several organs of the developing mouse embryo (E14.5-E16.5), including the lung bronchi. Haley et al*.* [223] found that RUNX1 is highly expressed in human fetal lung, predominantly in cartilage and epithelium. Tang et al*.* [224] observed RUNX1 expression in mouse respiratory epithelial and non-epithelial cells at E18.5, postnatal day 1 and 8 weeks. RUNX1 protein was 4.6-fold higher in the adult lung than at E18.5. Furthermore, the authors showed that conditional Runx1 deletion in respiratory epithelial cells led to a mild delay in lung maturation, with no impact in survival and postnatal lung maturation, but exhibiting increased respiratory distress, inflammation, and pro-inflammatory cytokines upon intra-tracheal LPS administration. Liang et al*.* [225] reported that during PH development in mice, endothelial precursor cells (EPCs) mobilize from the bone marrow into the circulation, and contribute to the remodeled pulmonary vasculature. Inhibition of endothelial-to-hematopoietic transition (EHT) by blocking Runx1 -a master regulator of EHT-, prevented disease progression in two experimental mouse models of PH. Lastly, they found high levels of Runx1 expression in circulating CD34^+^ CD133^+^ EPCs isolated from blood of PH patients, supporting the clinical relevance of this mechanism. In a follow-up study, they extended their observations to a more established rat model of PH, where they confirmed that Runx1 inhibition prevented and reverted PH. Finally, they showed that tissue-specific conditional deletion of Runx1 gene in adult endothelium or in pulmonary macrophages also protected the mice from developing experimental PH [226].

As extensively reviewed by Bush and Ivy [227], Down syndrome individuals have an enhanced frequency of pulmonary hypertension (PH), largely associated with congenital heart disease and persistent pulmonary hypertension of the newborn (PPHN); however, there likely are multiple other genetic contributions to such increased prevalence. While the molecular mechanisms underlying PH predisposition in Trisomy 21 remain to be fully elucidated, we speculate that RUNX1 overexpression and/or splicing imbalance could be a plausible contributing factor. Given that small-molecule inhibitors against RUNX1 have proven effective in experimental models of PH [226] and retinopathy (see section "Eye/endothelium"), future work should address their efficacy in the context of DS-associated PH.

#### Other cellular functions

Recent work from several groups has suggested critical roles for RUNX1 in Epithelial-Mesenchymal Transition-EMT-(recently reviewed by [228]), in DNA damage response (reviewed by [229]), in browning of white adipose tissue [230] and in diverse types of cancer (reviewed in [231]). The potential implications that DS-associated RUNX1 overexpression or alternative splicing deregulation could play in these paradigms are yet to be elucidated.

## Concluding remarks and future perspectives

**Fig. 3:**
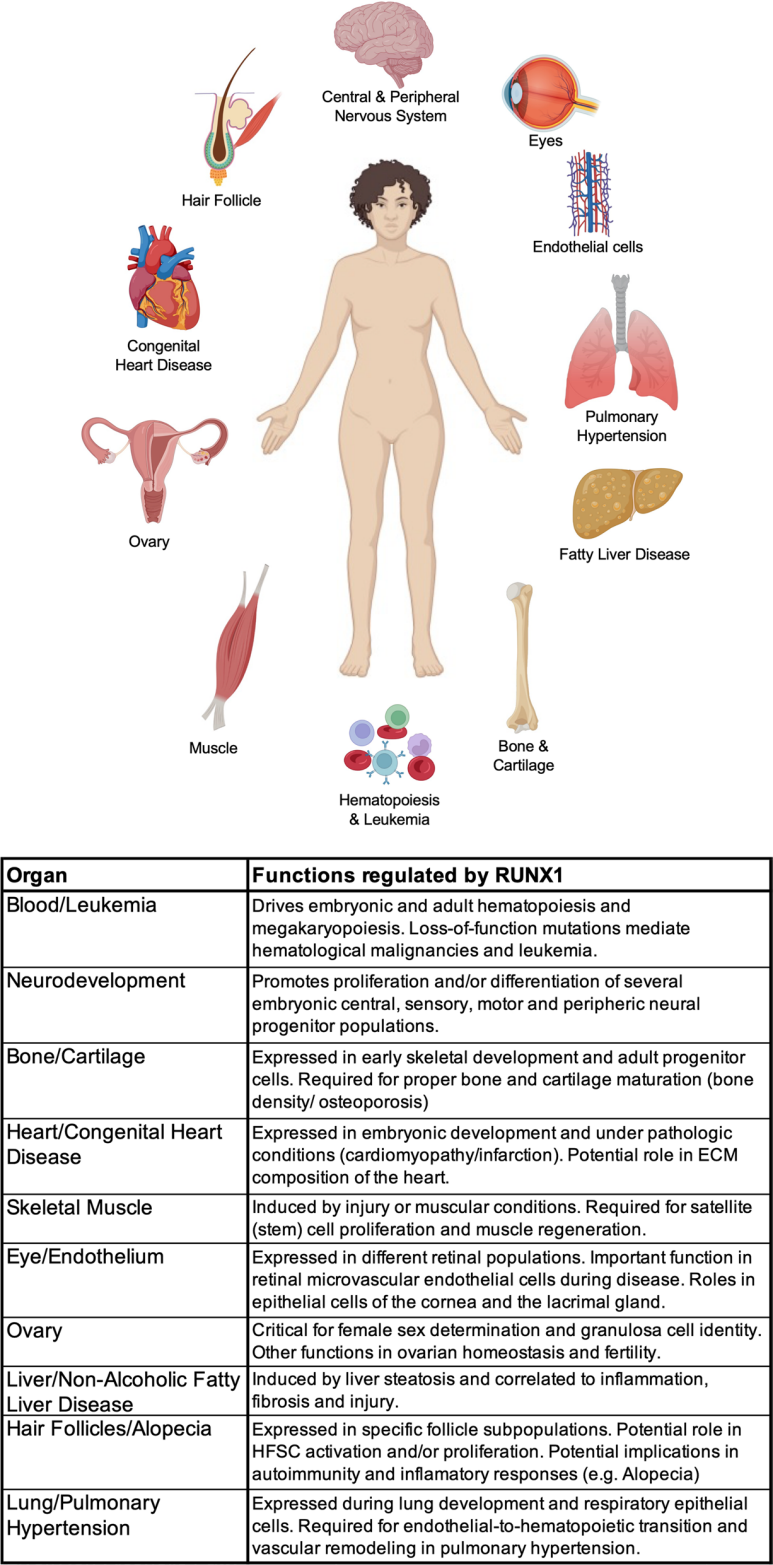
Summary of tissues, organs, and phenotypes where RUNX1 plays important developmental or homeostatic functions, and frequently altered or observed in individuals with Down syndrome and/or T21 model animals. Made with BioRender

While initially characterized as a master regulator of hematopoiesis and blood cell differentiation, RUNX1 is emerging as a critical transcription factor participating in a wide spectrum of developmental and homeostatic functions, most of which manifest frequent alterations in people with Down syndrome (summarized in Fig. 3). Although little is known about most of these ‘non-canonical’ RUNX1 roles across different tissues and organs in Trisomy 21, our review should set the stage for the research community to validate the many hypotheses proposed here. More importantly, the primate-specific alternative splicing that gives rise to RUNX1a should be taken into consideration when interpreting data from murine models. Finally, the fact that RUNX1 can be pharmacologically inhibited is an added motivation to test RUNX1 as a potential druggable target in specific pathological conditions associated with Down syndrome that currently have no treatment and represent important limitations in health and life quality for individuals with Trisomy 21 and their families.

## Data Availability

Not applicable.
